# Are we there yet? Australian road safety targets and road traffic crash fatalities

**DOI:** 10.1186/1471-2458-11-270

**Published:** 2011-04-29

**Authors:** Susan Gargett, Luke B Connelly, Son Nghiem

**Affiliations:** 1 Centre of National Research on Disability and Rehabilitation Medicine (CONROD), The University of Queensland, Edith Cavell Building, Royal Brisbane and Women's Hospital, Herston, Qld, 4029, Australia; 2 Australian Centre for Economic Research on Health (ACERH), The University of Queensland, Edith Cavell Building, Royal Brisbane and Women's Hospital, Herston, Qld, 4029, Australia

## Abstract

**Background:**

Road safety targets are widely used and provide a basis for evaluating progress in road safety outcomes against a quantified goal. In Australia, a reduction in fatalities from road traffic crashes (RTCs) is a public policy objective: a national target of no more than 5.6 fatalities per 100,000 population by 2010 was set in 2001. The purpose of this paper is to examine the progress Australia and its states and territories have made in reducing RTC fatalities, and to estimate when the 2010 target may be reached by the jurisdictions.

**Methods:**

Following a descriptive analysis, univariate time-series models estimate past trends in fatality rates over recent decades. Data for differing time periods are analysed and different trend specifications estimated. Preferred models were selected on the basis of statistical criteria and the period covered by the data. The results of preferred regressions are used to determine out-of-sample forecasts of when the national target may be attained by the jurisdictions. Though there are limitations with the time series approach used, inadequate data precluded the estimation of a full causal/structural model.

**Results:**

Statistically significant reductions in fatality rates since 1971 were found for all jurisdictions with the national rate decreasing on average, 3% per year since 1992. However the gains have varied across time and space, with percent changes in fatality rates ranging from an 8% increase in New South Wales 1972-1981 to a 46% decrease in Queensland 1982-1991. Based on an estimate of past trends, it is possible that the target set for 2010 may not be reached nationally, until 2016. Unsurprisingly, the analysis indicated a range of outcomes for the respective state/territory jurisdictions though these results should be interpreted with caution due to different assumptions and length of data.

**Conclusions:**

Results indicate that while Australia has been successful over recent decades in reducing RTC mortality, an important gap between aspirations and achievements remains. Moreover, unless there are fairly radical ("trend-breaking") changes in the factors that affect the incidence of RTC fatalities, deaths from RTCs are likely to remain above the national target in some areas of Australia, for years to come.

## Background

Death, injury and disability from road traffic crashes (RTCs) continue to be major global public health problems. Recent data suggest that the number of fatalities from RTCs is in excess of 1.25 million people per year with non-fatal injuries inflicted on a further 20 to 50 million people [1-3]. Globally they are the 10^th ^leading cause of death but in those aged 5-44 years they are one of the top three causes [3]. Disturbingly, it is predicted that by 2030 RTCs will have progressed to be the 5^th ^leading cause of death and that the number of people who will die annually from RTCs will have doubled from current levels [3]. The global economic cost of RTCs has been estimated at US$518b and has been calculated to account for 0.3%-4% of the gross national product (GNP) of various countries [4]. In Australia, RTCs have been estimated to cost approximately $18b per year [5,6]. The issue is of such widespread concern, the UN General Assembly has tabled a resolution proclaiming 2011-2020 to be the *Decade of Action for Road Safety *[7].

In efforts to address the issue numerous initiatives and strategies, with varying degrees of success, have been implemented. Broadly speaking, these have included legislative changes, enforcement activities, public educational programs, and vehicle safety and road engineering enhancements. Specific initiatives found to have been effective include the introduction of compulsory seat belts [8-10], reductions in speed limits and other speed-related measures such as the use of speed cameras [11-13], random breath testing [14,15], anti drink driving campaigns [16,17] and programs that target locations with higher than average crash rates (e.g., the Black Spot Program) [18]. A further approach that has been used increasingly in recent years has been the adoption of national road safety strategies that include specific targets for improvements in road safety outcomes. Indeed, the UN resolution includes the aim that nations set ambitious targets for the reduction in road fatalities by 2020. This study is concerned with evaluating the progress one country, Australia, has had in recent years in respect of reaching its road safety target.

The process of setting targets and the targets themselves have evolved substantially over time. Finland was perhaps the first country to establish a national road safety target. Its goal was to reduce the number of deaths from RTCs by 50%, from 1973 to the end of the 1970s [19]. Today the targets can be empirically-derived and based on quantitative modeling approaches that account for factors likely to affect the outcome, or aspirational goals determined through a top-down political process. Final outcomes such as reducing the number of deaths and/or serious injuries are most common but output or intermediate targets are also used [20]. For example, Canada's *Road Safety Vision 2010 *includes the sub-targets of a 20% reduction in fatalities and serious injuries from speed, and intersection-related crashes [21]. In Norway, targets have been set for the percent of adults using pedestrian reflective devices in the dark (70%), and the share of vehicle kilometers travelled (VKT) performed by cars with electronic stability control (95%), and enhanced neck protection devices (75%) [22]. Broader goals have also been specified. In New Zealand, an aim is to reduce the socio-economic cost of RTCs. By nominating this objective, society's willingness to pay for reducing the risk of road injury is incorporated into the decision-making process [23]. In Great Britain, welfare objectives for at-risk groups have been integrated. Their targets include a reduction in fatalities and serious injuries in children by 50%, and that casualties are reduced to a greater extent in disadvantaged neighbourhoods [20]. Alternatively, some jurisdictions, most notably Sweden, have set visions for road safety with the definitive aim of a zero tolerance of deaths and serious injuries from RTCs [24]. The most recently adopted road safety strategy in Australia, *Towards Zero - Road Safety Strategy *[25], endorsed by the Western Australia Government in 2009, also articulates this ultimate aim.

The benefits of road safety targets are widely advocated. The OECD [26] argues that targets may improve road safety by; encouraging the development of enhanced or more realistic road safety programs (that can, in turn, lead to a more efficient use of scarce public resources), communicating the importance of road safety to people who can affect it, giving direction to policy-making and motivating stakeholders to act, and by holding managers of road transport systems to account [20,26]. Others have suggested that target-setting that results in the monitoring of outcomes can give rise to early warning signs of potential problems, thereby enabling alternative strategies (such as additional funding or a change in approach) to be considered in a timely fashion [27].

Sub-national targets are also supported on the basis that they may widen the sense of responsibility and result in more partnerships being established and greater action [20]. This idea is compatible with the notion, in economics, of public goods (which are non-rival and non-exclusive in consumption) and the attendant "free-rider" problem. Establishing responsibility, at the local level, for the achievement of targets may prevent free-riding and thereby lead to a more efficient social outcome.

The putative benefits of ambitious long-term aspirational goals are that the media's and public's awareness of the issue is raised and thus, politicians may be motivated 'to support proposed policy and legislative changes and allocate sufficient resources to major problem areas'. Additionally, such goals may serve to alter the community's view of the inevitability of road trauma [20]. However, there have also been criticisms of the use of such targets. It has been suggested that a possible outcome could be that the resulting policy focus may be distorted if areas where progress can be easily measured are emphasised at the expense of other areas [27]. It is also possible that if aspirational targets are radically divorced from what is actually achievable or achieved, they may come to be regarded as irrelevant or, worse, demotivating.

To date, relatively few studies have evaluated the impact of RTC targets but available evidence suggests that target-setting is correlated with improvements in RTC casualty rates. Elvik [28] reported that Norwegian counties with more ambitious targets succeeded in reducing the accident rate per kilometer traveled, to a greater extent than counties with less ambitious or non-quantified targets. Wong et al. [29] conducted a before-and-after analysis of 14 countries and found a significant reduction in fatalities after a quantified target was set.

Though Australia's road toll remains high, considerable improvements have been achieved over recent decades. RTC fatalities in Australia have decreased from a peak of almost 3,800 in 1970 to just over 1,500 in 2009 [30,31]. Australia's (1^st^) *National Road Safety Strategy *covered the period 1992-2001 and included the target, 'less than 10 deaths per 100,000 population by 2001' [32]. The target specified in the (2^nd^) *National Road Safety Strategy 2001-2010 *(*NRSS 2001-2010*), is for 'no more than 5.6 fatalities per 100,000 by 2010' [33]. A national RTC injury reduction target was not set for Australia as an appropriate national injury database from which necessary benchmark information could be obtained, did not exist.

Constitutionally, Australia is made up of six states; New South Wales (NSW), Victoria (Vic.), Queensland (Qld), South Australia (SA), Western Australia (WA), and Tasmania (Tas.) and two territories, the Australian Capital Territory (ACT) and Northern Territory (NT). The public responsibility for road safety is shared across the three levels of government in Australia; national, state/territory, and local. While public road safety initiatives have been coordinated nationally since the adoption of the (1^st^) *National Road Safety Strategy *in 1992 [34], road safety policy and enforcement activities are principally driven, implemented, and enforced by state and territory governments. Across Australia there is substantial variation in RTC outcomes and in the factors that affect RTC rates (e.g., government policy, law enforcement activities and geographical variables). Interestingly, each state and territory has its own road safety strategy and most also specify a state/territory-specific target for reducing fatalities. Presumably these have been developed due to the varied challenges the different jurisdictions face in relation to road safety. Additionally, respective state and territory governments may decide that the national target is either too ambitious or not sufficiently ambitious for their own jurisdictions. Though details on how the target rate for Australia was established have not been published, presumably intra-state variations were taken into account when the national target was set. Furthermore, we expect these spatial variations to persist over time and that fatality rates in some jurisdictions will remain above the national target while others will be driven below it.

The first widely-cited paper on statistical modeling of RTC fatalities was by Smeed in 1949 [35]. Though his formula relating fatalities to traffic volume (i.e., number of vehicles) and the population has been unable to explain changes in RTC fatality rates over time [36,37], his work provided the foundation for an area of research that has grown and developed in magnitude and importance. A substantial literature on the topic now exists. While some structural models of RTC rates have included only measures of exposure and risk [37,38], most use a range of socio-demographic, economic, environmental, and policy-related variables with the aim of describing, explaining and/or predicting road safety outcomes [39-42]. Critical reviews of the factors that influence RTC rates, and aggregate models of RTCs *per se *have also been undertaken [43]. Modeling approaches have also progressed with the application of sophisticated state-space-time series models [10,44].

The aim of this analysis is to evaluate Australia's progress towards its national fatality rate target on the basis of past trends. Derived empirical estimates of the trends are reported and used to determine projections of when the target may be achieved. The analysis is also conducted at a disaggregated level with trends in the fatality rates in each state and territory estimated, and used to indicate the progress the individual jurisdictions have made towards the national target. While it is a national (rather than sub-jurisdictional) target and although it may be achieved without all jurisdictions attaining it at the same time, it provides a uniform benchmark against which the outcomes of the respective states and territories can be compared. As such, the state/territory analyses serve to highlight the regional variations that exist. A general descriptive analysis of fatality rates is also conducted. The analysis does not seek to establish the structural determinants of RTCs and does not include hypothesized causative factors in the models. Nor does it address the question of whether or not specifying a target is an effective way to reduce the road toll. Rather, it relies on the assumption that recent trends in RTC fatalities will continue in the near term, and simply examines the rate at which Australia and its jurisdictions are approaching the target to which policy-makers have aspired.

Although using past observations to predict future outcomes is a standard forecasting technique that is widely-used in policy circles, the limitations of the approach are important to consider. Projections based on past trends implicitly assume that past behaviours, relationships, and outcomes will continue unchanged over the forecasting horizon however, in relation to RTCs this has not always been the case [45,46]. In an effort to address this, an important consideration in the analysis has been the careful choice of the time-series data such that contemporary rather than archaic trends in RTC outcomes are used in determining the forecasts. A related issue is whether a model incorporating factors known/hypothesized to impact on RTC outcomes (such as e.g., legislations, car safety technology and road engineering) would have been more appropriate than a pure time series methodology. While a structural model may have had greater predictive power [43] the authors believe that existing data limitations and estimation issues were likely to have compromised such an analysis for Australia.

The national road safety target is used in Australia as part of the overall strategy to reduce the burden of road trauma. The rationale for this study is that for a target to have relevance, progress towards it must be assessed and in relation to road safety targets, ideally, publicly examined. At the system level such information may be useful to those agencies that are charged with pursuing improvements in road safety, e.g. to reassess and reprioritize their approach if outcomes are falling short of expectations. Conversely, strategies that have resulted in successful outcomes can be considered for implementation elsewhere. Such objectives may be facilitated if the analysis occurs at a disaggregated and aggregated level. In addition, increased awareness of the target and progress towards it may enhance public acceptance of road safety initiatives and could play a small part in promoting positive behavioural changes in individuals, who, ultimately, may have the strongest marginal influence on road safety.

## Methods

Annual fatality rate data i.e., deaths from RTCs per 100,000 population for Australia and its six states and two territories for 1971-2009, were used in this study. Data for 1971-2007, and for 2008-2009 are from the Bureau of Infrastructure Transport and Regional Economics (BITRE) [30,47], respectively. BITRE is an entity within the Australian Government's Department of Infrastructure and Transport that compiles the road safety and road incident data supplied by police agencies to the state and territory road safety authorities. Although concerns about the accuracy of police-reported road safety outcome data have been raised [48-50] these have mostly concerned the problem that injury (i.e., non-fatality) crashes tend to be under-reported and the question of whether attempts to use such data to describe injury epidemiology following RTCs generates substantial measurement error in relation to injury severity, in particular. RTC fatality rate data, by comparison, are not subject to these limitations. In order to compute population fatality rates for 2008 and 2009 we used Estimated Resident Population data supplied by the Australian Bureau of Statistics [51]. We note that RTC data for 2009 are preliminary.

The study proceeded in several steps. First, some general descriptive data were determined. Changes in the fatality rates over the past four decades were estimated by calculating percentage changes in the rates from the first to last year of each of four consecutive decades/periods. Estimates of the number of fatalities that would result in each jurisdiction achieving the national target in 2010 were calculated. This was done to enable a comparison of recent outcomes, specifically the average annual number of fatalities per jurisdiction 2007-2009, with the number of fatalities projected by the national target for that jurisdiction in 2010. A comparison between the state/territory-specific targets and recent outcomes in the respective jurisdictions has also been conducted. However this examination is limited, as in some instances the manner in which the state/territory-specific target has been specified and/or its timeframe restrict useful comparisons.

Next, various univariate models of past trends in fatality rates were estimated. Two issues were addressed prior to estimation of the models, the time-span over which the trends were estimated and model specification. Initially three periods were considered: 2001-2009, 1992-2009, and 1971-2009. The period 2001-2009 corresponds to the years over which the *NRSS 2001-2010 *has been in operation (at the time of writing). Thus, it could be argued that as a consistent national road safety policy agenda prevailed over this period this is an appropriate time-span for the analysis, as one factor (i.e., the national road safety policy agenda) that may influence RTC outcomes has (theoretically) been constant over the period. However, this option was not used as nine observations were considered too few to produce reliable estimates of past trends. Elvik [52] has also argued that a period of less than 10 years is too short for identifying long-term trends in road safety outcomes. The second option, 1992-2009, represents the years over which either the 1^st ^or 2^nd ^national strategy has operated. This is our preferred time period for the analysis for two reasons. First (and as for 2001-2009), the road safety agenda was coordinated nationally over this period. Second, the number of observations and the time-span covered by these data are considered sufficient to enable estimates of past trends to be determined with some confidence. Trends have also been estimated using data for 1971-2009 but mostly for comparative purposes only. Although these estimations have the advantage of a greater number of observations, road safety outcomes have changed substantially over this period in Australia and trends estimated from 1971 are unlikely to be as representative of future outcomes as those determined using more recent data. Only data from 1971 were considered as RTC fatalities in Australia peaked in 1970 and there has been a fairly consistent downward trend in the number of fatalities since that time.

In determining appropriate trend models to estimate and following Elvik [45], various specifications were considered. The overall reduction in fatality rates since the 1970s and the more recent phenomenon of a gradual decrease in the rate of this reduction were important considerations. As well, it was judged that it would be unlikely for RTC fatalities to be completely eliminated in the foreseeable future or that there would be an appreciable or persistent increase in fatality rates in the short-term. However, the possibility of an increase in fatality rates can not be entirely dismissed as factors that escalate the population's exposure to the risk of a RTC (e.g., the level of motorization and VKT) continue to increase. Linear and logarithmic models were discounted as these may forecast negative rates in future years. Quadratic trend models were also rejected as these may have forecasted either continual increases in the rates or negative rates. Only power (or geometric) and exponential trend models were estimated as these met our *a priori *assumption of a continued but slowing decrease in RTC fatality rates in the short-term. (It is noted however, that these models imply that the realisation of 'Vision Zero' in Australia is beyond the foreseeable timeframe.)

Both specifications were used to estimate models for each jurisdiction over both time periods (i.e., 1992-2009 and 1971-2009). The equations estimated using ordinary least squares (OLS), were:

where *y *indicates the natural log of the annual fatality rate, *α *indicates the log of the intercept, *x *indicates time (year) and *β *is the time trend parameter to be estimated. The results of the alternative specifications were compared to determine the preferred options which were selected on the basis of a range of summary statistics including *R^2 ^*values, Akaike information criterion (AIC) and the sum of squared residuals.

The results of the preferred models were then used to create out-of-sample forecasts to derive indications of when the national fatality rate target is likely to be achieved by the different jurisdictions. These predictions are constrained because of the limited number of observations used in the regressions, the absence of explanatory variables in the models apart from a time trend, and the uncertainty associated with predicting road safety outcomes [53]. Forecasts were ceased when the target rate was reached or after ten predicted values had been generated if the target rate had not been reached (i.e., projections beyond a ten-year horizon were not made). Confidence intervals (95%) were estimated to evaluate the likely precision of the forecasts.

The use of OLS regression to estimate the trends needs further discussion. The issue of the non-normal errors in models of RTC rates (estimated using OLS and/or other techniques) has been discussed in the literature [54-57]. The problem relates to the data being analysed. RTC rates are count data that take only non-negative values. Count data are typically modelled using a Poisson regression or one of its a derivates, negative binomial or zero-inflated models, collectively referred to as generalized linear models (GLM). This approach has not been used here but standard diagnostic checks of the residuals have been conducted and where evidence of non-normal residuals was detected the models were disregarded.

## Results

### General descriptive analyses

**Figure 1:**
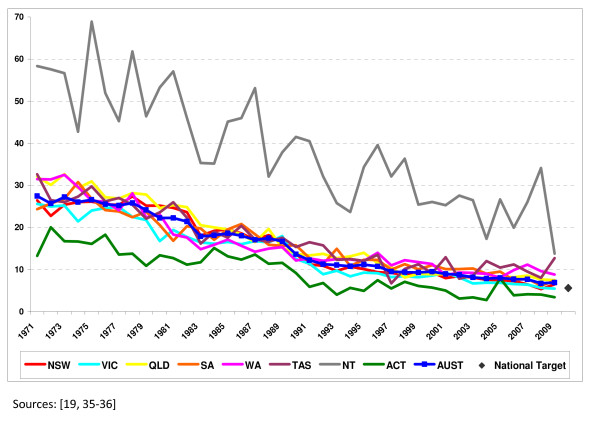
**RTC fatalities per 100,000 population, Australia and Australian states and territories, 1971-2009**.

Annual fatality rates for Australia and its eight constituent jurisdictions are presented in Table 1 and Figure 1. The substantial decrease in the rates that has occurred in all Australian jurisdictions over the past 40 years and a general slowing in this decrease in most jurisdictions since the early 1990s are both clearly evident. Differences in the rates across the jurisdictions are also apparent with two outlier jurisdictions, the NT and ACT, having the highest and lowest rates respectively. In relation to progress towards the target of no more than 5.6 deaths per 100,000, casual empiricism suggests that while some of the jurisdictions appear to be well-placed to meet the target, or indeed have already done so, others are not. Specifically, the rate for Australia in 2009 was 6.9 deaths per 100,000. Encouragingly, two jurisdictions, the ACT and Vic., have already achieved a rate below the target with the rate in the ACT having been less than the target rate in all years since 2001 except one. In contrast, since the commencement of the *NRSS 2001-2010 *the rate in the NT has been, on average, four times greater than the target rate even though it recorded its lowest RTC fatality rate in the past forty years (13.8 deaths per 100,000) in 2009.

**Table 1:** Annual RTC fatality rate data per 100,000 population: Australia and Australian states and territories, 1971-2009

	Australia	NSW	Vic.	Qld	SA	WA	Tas.	ACT	NT
1971	27.47	26.43	25.63	32.08	24.33	31.5	32.66	13.23	58.32
1972	25.72	22.77	24.99	30.13	25.69	31.42	26.48	20.03	57.56
1973	27.24	25.40	25.22	32.69	26.78	32.51	26.05	16.73	56.63
1974	26.03	26.05	21.46	29.33	30.77	29.62	27.33	16.65	42.75
1975	26.59	26.12	24.03	30.96	26.79	26.32	29.75	16.08	68.91
1976	25.53	25.49	24.62	27.19	24.1	26.14	26.19	18.29	51.92
1977	25.21	25.35	24.86	26.86	23.79	24.08	26.99	13.57	45.22
1978	25.8	27.39	22.49	28.18	22.45	28.1	25.38	13.76	61.83
1979	24.17	25.20	21.77	27.81	23.75	22.38	22.1	10.87	46.43
1980	22.27	25.20	16.78	24.58	20.56	23.09	23.61	13.38	53.28
1981	22.25	24.66	19.41	25.33	16.83	18.31	25.98	12.74	57.09
1982	21.42	23.63	17.76	24.83	20.28	17.63	22.33	11.16	46.04
1983	17.9	18.05	16.45	20.55	19.77	14.83	16.17	11.72	35.32
1984	18.11	19.19	16.12	20.01	17.06	15.89	18.96	15.1	35.17
1985	18.63	19.53	16.58	19.52	19.54	17.13	17.61	13.13	45.11
1986	18.03	18.60	16.05	18.33	20.83	15.63	20.38	12.36	45.98
1987	17.04	17.07	16.75	16.52	18.38	14.24	17.14	13.56	53.1
1988	17.46	18.17	16.45	19.67	15.87	14.98	16.62	11.39	32.07
1989	16.66	16.62	17.96	15.14	15.64	15.33	17.57	11.58	37.85
1990	13.66	13.66	12.52	13.76	15.78	12.15	15.36	9.21	41.53
1991	12.23	11.24	11.38	13.34	12.72	12.65	16.5	5.88	40.49
1992	11.28	10.88	8.89	13.73	11.33	12.06	15.75	6.79	32.13
1993	11.05	9.68	9.73	12.73	14.92	12.46	12.3	4.01	25.77
1994	10.8	10.66	8.4	13.12	10.84	12.39	12.48	5.64	23.65
1995	11.16	10.12	9.25	13.97	12.32	12.05	12.03	4.92	34.36
1996	10.76	9.36	9.14	11.53	12.28	13.99	13.49	7.46	39.59
1997	9.54	9.18	8.2	10.6	9.99	10.97	6.76	5.5	32.1
1998	9.38	8.77	8.41	8.09	11.28	12.23	10.17	7.1	36.34
1999	9.32	9.00	8.17	8.97	10.08	11.79	11.24	6.08	25.42
2000	9.49	9.30	8.58	8.9	11.03	11.31	9.12	5.71	26.08
2001	8.95	7.97	9.24	8.93	10.12	8.68	12.93	5.01	25.28
2002	8.73	8.46	8.16	8.67	10.12	9.3	7.83	3.1	27.58
2003	8.15	8.08	6.7	8.14	10.25	9.22	8.58	3.38	26.49
2004	7.86	7.60	6.88	7.97	9.02	8.98	12.01	2.75	17.31
2005	7.98	7.52	6.85	8.26	9.53	8.08	10.49	7.87	26.64
2006	7.72	7.28	6.57	8.19	7.46	9.86	11.23	3.89	19.94
2007	7.69	6.49	6.42	8.58	7.83	11.16	9.53	4.12	26.05
2008	6.73	5.35	5.7	7.64	6.17	9.63	8.04	4.05	34.12
2009	6.9	6.49	5.44	7.49	7.33	8.81	12.73	3.42	13.79

Sources: [30,47]

Table 2 presents descriptive statistics for 1971-2009 and 2001-2009. For 1971-2009, fatality rates have ranged from a high of nearly 69 deaths per 100,000 in the NT in 1975 to less than 3 per 100,000 (2.8) in the ACT in 2004. The mean fatality rate for Australia over this period was 15.7 per 100,000, though since the *NRSS 2001-2010 *has been in effect it has reduced to less than 8 per 100,000. Consistent with these improvements, in all jurisdictions the highest annual fatality rate occurred in the 1970s whereas the lowest rate in six of the nine jurisdictions occurred in either 2008 or 2009. The exceptions are Tas., ACT and WA which recorded their lowest fatality rate since 1970 in 1997, 2004, and 2005, respectively.

**Table 2:** Descriptive statistics of fatalities per 100,000: Australian states and territories 1971-2009 and 2001-2009

*1971-2009*	Australia	NSW	Vic.	Qld	SA	WA	Tas.	ACT	NT
Mean	15.7	15.6	14.1	17.2	16	16.3	17.1	9.5	38.6
Median	13.7	13.7	12.5	14	15.6	14	16.2	9.2	36
Maximum	27.5	27.4	25.6	32.7	30.8	32.5	32.7	20	68.9
Minimum	6.7	5.4	5.4	7.5	6.2	8.1	6.8	2.8	13.8
Std. Dev.	7.2	7.6	6.7	8.5	6.6	7.3	7.1	4.9	13.4

*2001-2009*									

Mean	7.9	7.2	6.9	8.2	8.6	9.3	10.4	4.2	24.1
Median	7.9	7.5	6.7	8.2	9.0	9.2	10.5	3.9	26.1
Maximum	9	8.5	9.2	8.9	10.3	11.2	12.9	7.9	34.1
Minimum	6.7	5.4	5.4	7.5	6.2	8.1	7.8	2.8	13.8
Std. Dev.	0.7	1.0	1.2	0.5	1.5	0.9	2.0	1.5	6.1

In Table 3 fatality rate reductions for each jurisdiction in four successive decades/periods are given. In determining relevant periods for comparison, the decades/periods 1992-2001 and 2001-2009 were used as they coincided with the periods over which the national road safety strategies have operated. The periods, 1972-1981 and 1982-1991, were determined so that data for four periods of approximately equal length could be compared. To explain the data, the top left-hand-side cell in the table indicates that RTC fatality rates in Australia fell by 13.2% between 1972 and 1981. Data in parentheses indicate an increase in the rate over the period. The data indicate that for all but two jurisdictions, Vic. and NT, the period over which the greatest percentage reduction occurred was either the 1970s or 1980s decades/periods, whereas in five jurisdictions the smallest percentage reduction occurred in the 1990s or 2000s decades/periods. Only one jurisdiction (entirely) defied this general picture: in the NT the percentage reductions has successively increased in magnitude over the four periods. It is important to note however, that the data in Table 3 are based on point data i.e., data for a specific year and thus, may give a distorted impression of past outcomes due to either large fluctuations or outlier values, unrepresentative of the general trend over the period, being used in the estimations. This issue is perhaps more likely in jurisdictions with small populations and the data for the NT for 2001-2009 highlight the issue. The rates in the NT in 2001 and 2009 were 25.3 and 13.8 fatalities per 100,000 respectively, which represents a 45.5% reduction over the period. However, if the rate for 2008 had been used instead of that for 2009 a 35% increase in the rate would have been recorded.

**Table 3:** Percentage reductions in fatality rates Australian states and territories

	Australia	NSW	Vic.	Qld	SA	WA	Tas.	ACT	NT
1972 to 1981	13.2	(8.3)	22.4	15.9	34.6	41.7	1.9	36.5	0.9
1982 to 1991	43.0	52.5	36.0	46.4	37.4	27.8	26.0	47.3	12.0
1992 to 2001	20.7	26.7	(3.9)	35.0	10.7	28.0	17.9	26.2	21.3
2001 to 2009	22.9	18.6	41.1	16.1	27.6	(1.5)	1.5	31.7	45.5

Notes: Data in parentheses indicate increases in fatality rates rather than reductions.

Progress towards the target has also been considered by comparing the number of fatalities in a jurisdiction in recent years with the number of fatalities implied by the target rate. Table 4 presents the estimated maximum number of fatalities per jurisdiction that would not breach the target of 5.6 deaths per 100,000 and thus, would equate with the jurisdiction achieving the target in 2010. These were calculated using estimates of the jurisdictions' populations in June 2010, and the target rate. For comparative purposes, the average annual number of fatalities for each jurisdiction 2007-2009 is also given. Differences between the recent outcomes and the predicted number of deaths that would enable the target to be met are reported as percentages in the final column of the table and are substantial in most cases. For instance, approximately 17% fewer fatalities would need to occur in Australia in 2010 than have occurred annually, on average, over the past three years for the target to be met. Parentheses have been used where the average number of fatalities 2007-2009 has been less than the predicted target number for 2010. This is the case for the two jurisdictions that have already achieved the target rate, i.e., the ACT and Vic.

**Table 4:** Estimated number of fatalities by jurisdiction that would enable the target to be met in 2010

	**Projected Population June 2010**^**a**^	Estimated number of fatalities equal to 5.6 fatalities per 100,000 population in 2010	Average annual number of fatalities, 2007-2009	**Percentage difference between average annual fatalities, 2007-2009, and estimated target (maximum) number of fatalities in 2010**^**b**^
Aust.	22,410,783	1,255	1,518	17
NSW	7,254,446	406	423	4
Vic.	5,563,364	312	310	(0.5)
Qld	4,538,829	254	339	25
SA	1,645,305	92	114	19
WA	2,308,890	129	214	40
Tas.	508,240	28	50	43
ACT	358,590	20	13	(55)
NT	230,942	13	55	77

Notes: ^a ^Population projections per jurisdiction for June 2010 have been estimated using data on 'estimated resident populations' for September and December 2008, and March, June and September 2009 [68]. Percentage changes in these data were calculated and the average quarterly percent changes from September 2008 to September 2009 were determined. These were applied to the respective population estimates for September 2009 to calculate population estimates for December 2009. To calculate population estimates for June 2010, this process was repeated with the respective average quarterly percent changes sequentially applied to the newly-derived population estimates for December 2009, and then for March 2010. Projected population estimates for the various jurisdictions for June 2010 were published in 2008 [69] but recent estimates of the estimated resident populations for September 2009, published in March 2010, have, in some cases, surpassed these earlier estimates for June 2010. As it is not anticipated that the populations will reduce in the near-term, these alternative estimates have been calculated and used (as outlined), in preference to the estimates published in 2008. ^b ^The percentage difference data in parentheses indicate that the average number of annual fatalities, based on data for 2007 to 2009, is less than the estimated number of fatalities in 2010 that would meet the national target rate.

In order to consider the achievements of each state and territory against their respective government's road safety objectives, the state/territory-specific RTC strategies and state/territory-specific RTC target/s are summarized in Table 5. In the final column, recent outcomes are given and contrasted with respective state/territory-specific targets, where possible. Though these comparisons are limited and incomplete, it is clear that for some jurisdictions a sizeable gap between recent outcomes and the specified target still exist.

**Table 5:** Australian state and territory road safety strategies and targets

	Strategy	Targets	Comments
NSW	*Road Safety 2010*	halve the road toll, saving 2,000 lives by 2010	• reference year = 1998 during which there were 556 RTC fatalities • thus, target is assumed to be no more than 253 fatalities in 2010 • average no. of fatalities 2007-2009 = 423

Vic.	*Arrive Alive 2008-2017*	save an extra 100 lives per year by the end of 2017, prevent over 2,000 serious injuries per year and reduce the severity of serious injuries	• reference year (assumed to be) 2007 during which there were 332 fatalities • thus, target assumed to be no more than 232 fatalities in 2017

Qld	*Queensland Road Safety Strategy 2004-2011* *'safe4life'*	less than 5.6 deaths per 100,000 by 2011	• fatality rate 2009 = 7.5 per 100,000

SA	*The South Australian Road Safety Strategy 2003-2010*	does not identify a state-specific target but refers to the national target of 5.6 deaths per 100,000 by the end of 2010	• fatality rate 2009 = 7.3 per 100,000

WA	*Towards Zero: getting there together 2008-2020*	11,000 fewer people killed or seriously injured by 2020 (if strategy fully implemented) • represents a 40% ↓ on the average number of people killed or seriously injured each year between 2005 & 2007	

Tas.	*Tasmanian Road Safety Strategy 2007-2016*	a 20% ↓ by 2010 in serious injuries & fatalities from 2005 a 20% ↓ by 2015 in serious injuries & fatalities from 2010 a 20% ↓ by 2020 in serious injuries & fatalities from 2015	• reference year = 2005 during which there were 51 fatalities • a 20% ↓ would be ≈ no more than 41 fatalities in 2010 • average no. of fatalities 2007-2009 = 50

ACT	*ACT Road Safety Strategy 2007-2010*	achieve better than the national target of 5.6 fatalities per 100,000	• fatality rate 2009 = 3.4 per 100,000

NT	*Northern Territory Road Safety Strategy 2004-2010*	no more than 15 fatalities per 100,000 in 2010 • represents more than a 40% ↓ on current levels	• reference year = 2003 during which there were 26.5 fatalities per 100,000 • fatality rate 2009 = 13.8 per 100,000; • however, average fatality rate 2007-2009 = 24.7 per 100,000

Sources: [25,70-76]

### Univariate trend analyses

Results of the univariate trend models are given in Table 6. In all instances the criteria used for model selection led to the same preferred specification for each jurisdiction in both time periods. Exponential trends were selected as the preferred model for Australia, NSW, Vic., SA, ACT, and NT whereas power or geometric trends were preferred for Qld, WA and Tas. As previously noted, our initial preference was to use 1992-2009 data for the estimations. This was done for all but two jurisdictions, NSW and Tas. A suitable specification was not determined for Tas. as no statistically significant time trend was found for 1992-2009, and the equation estimated using 1971-2009 data was not statistically robust (i.e., the residuals were not normally distributed). Similarly for NSW, non-normality of the residuals from the equation estimated using 1992-2009 data meant that the estimation using 1971-2009 data was preferred. The preferred specification for each jurisdiction is indicated in Table 6. Serial correlation of the error terms was present in some of the regressions. To correct for this, serial correlation-robust standard errors were estimated using the Newey-West heteroscedastic and autocorrelation consistent (HAC) covariance estimator [58].

**Table 6:** Regression results by jurisdiction for trends in annual fatality rates: 1971-2009 and 1992-2009

*1971-2009*	Preferred Model	**Variable**^**a**^	Coefficient	**Std. Error**^**b**^	*t-*stat	***R***^***2***^	**SE**^**c**^	**BG**^**d**^	**J-B**^**e**^
Australia^(+)^	exponential	*α*	84.05	3.03	27.75***	0.97	0.078	18.86***	1.71
		*x*	-0.04	0.002	-27.92***				

NSW^(■ +)^	exponential	*α*	90.53	5.45	16.62***	0.94	0.125	17.85***	1.28
		*x*	-0.04	0.00	-16.20***				

Vic.^(+)^	exponential	*α*	87.58	2.88	30.37***	0.95	0.11	11.32***	0.95
		*x*	-0.04	0.00	-29.51***				

Qld^(+)^	power	*α*	662.77	30.73	21.56***	0.97	0.095	9.3***	1.29
		*lnx*	-86.90	4.05	-21.47***				

SA	exponential	*α*	75.16	2.93	25.68***	0.94	0.103	0.13	1.33
		*x*	-0.04	0.001	-24.76***				

WA	power	*α*	525.37	45.83	11.46***	0.91	0.128	12.75***	0.92
		*lnx*	-68.81	6.03	-11.40***				

Tas.	power	*α*	519.00	37.03	14.02***	0.84	0.172	0.13	20.43***
		*lnx*	-67.96	4.88	-13.94***				

ACT	exponential	*α*	92.75	7.10	13.06***	0.81	0.25	1.85	0.26
		*x*	-0.05	0.00	-12.76***				

NT	exponential	*α*	59.01	5.52	10.70***	0.73	0.195	0.58	0.30
		*x*	-0.03	0.002	-10.05***				

*1992-2009*									

Australia^(■)^	exponential	*α*	62.6	3.09	20.29***	0.96	0.034	0.05	0.73
		*x*	-0.03	0.001	-19.58***				

NSW	exponential	*α*	67.76	6.25	10.85***	0.87	0.069	0.05	9.74***
		*x*	-0.03	0.003	-10.51***				

Vic.^(■)^	exponential	*α*	60.92	7.36	8.27***	0.80	0.08	2.19	1.33
		*x*	-0.03	0.00	-8.00***				

Qld^(■ +)^	power	*α*	542.62	83.62	6.49***	0.78	0.102	5.86**	1.38
		*lnx*	-71.09	11.00	-6.46***				

SA^(■)^	exponential	*α*	72.4	9.77	7.41***	0.76	0.107	1.2	1.28
		*x*	-0.04	0.004	-7.18***				

WA^(■)^	power	*α*	339.80	74.91	4.54***	0.56	0.108	1.5	0.19
		*lnx*	-44.39	9.86	-4.50***				

Tas.	power	*α*	223.70	146.77	1.52	0.12	0.212	0.74	1.72
		*lnx*	-29.12	19.31	-1.51				

ACT^(■)^	exponential	*α*	60.22	25.88	2.33**	0.24	0.28	0.03	0.11
		*x*	-0.03	0.01	-2.27**				

NT^(■)^	exponential	*α*	53.15	21.24	2.50**	0.26	0.234	0.5	0.21
		*x*	-0.02	0.01	-2.35**				

Notes: ^a ^α indicates the natural logarithm of the constant; ^b ^Std. Error indicates the standard error of the estimator; ^c ^SE is the standard error of the regression; ^d ^BG is the Obs* R-squared statistic from the Breusch-Godfrey Lagrange multiplier test for serial correlation (1 lag); ^e ^J-B is the statistic from the Jarque-Bera test for the normality of the residuals. The preferred model for each jurisdiction is indicated by ^(■) ^and ^(+) ^indicates the equation has been estimated using the Newey-West heteroscedasticity and autocorrelation consistent (HAC) covariance estimator. ***, ** and * indicate statistical significance at 1, 5, and 10 percent respectively. Other abbreviations are as defined in the text.

The results show that since 1992 there has been a significant decrease in the national fatality rate that has averaged 3% per year. Reductions were also statistically significant in all states and territories since 1992 except Tas., and ranged from 3.6% per year in Qld to 2.2% in WA. The estimated coefficients of the exponential trend variable provide direct measures of the average annual percentage change in fatality rates. The coefficients from the geometric (or power) trend equations are estimates of the elasticity value at the mean. These have been converted into average annual percent changes by dividing the estimated coefficient by 1% of the mean value of the trend variable (e.g., for Qld 1992-2009; 71.09/20 = 3.55 where 20 = 1% of the mean value of the trend variable). Clearly these outcomes are of great practical significance.

In relation to the data for 1971-2009, the decrease was significant in all jurisdictions though the results for Tas. are not statistically robust (and therefore disregarded). The average decrease across Australia over this period was 4.1% per year, and ranged from 4.6% in the ACT to 2.8% in the NT. For most of the jurisdictions the preferred models have high explanatory power especially when estimated using the longer time series. However, in the jurisdictions with the smallest populations i.e., the two territories, since 1992 time trends explain only approximately a quarter of the variation in the rates. As stated, the factors that have contributed to these reductions are not the subject of this analysis.

Table 7 presents our best projections of the years in which the national target may be reached. Greatest confidence may be placed in the forecasts for Australia given the estimated confidence intervals. Estimates for the ACT and Vic. are not presented as these jurisdictions have already achieved the target rate. Estimates for Tas. are not presented due to our lack of confidence in the models estimated for this jurisdiction. The calculations project that the target rate may be reached nationally in 2016 and around this time in Qld and SA, but substantially earlier in NSW (2011), Australia's most populous state. However, unless there are considerable changes to the trends in WA and the NT, the national target may not be reached in these jurisdictions for a considerable period. The latter results suggest that considerable efforts are still required, particularly in these jurisdictions, to hasten reductions in RTC fatalities.

**Table 7:** Predicted fatality rates by jurisdiction

	Australia		NSW		Qld		SA		WA		Tas.		NT	
	**Fatality** **Rate**	**95%** **CIs**	**Fatality** **Rate**	**95%** **CIs**	**Fatality** **Rate**	**95%** **CIs**	**Fatality** **Rate**	**95%** **CIs**	**Fatality** **Rate**	**95%** **CIs**	**Fatality** **Rate**	**95%** **CIs**	**Fatality** **Rate**	**95%** **CIs**

*2009*	*6.9*		*6.5*		*7.5*		*7.3*		*8.8*		*12.7*		*13.8*	
2010	6.7	6.3-7.2	5.7	4.4-7.3	6.8	5.5-8.5	7.1	5.6-8.9	8.6	6.8-10.8	8.0	5.6-11.3	21.0	12.8-34.4
2011	6.5	6.1-7.0	5.4	4.2-7.0	6.6	5.3-8.2	6.8	5.5-8.6	8.4	6.7-10.6	7.7	5.4-10.9	20.4	12.5-33.5
2012	6.3	5.9-6.8			6.3	5.1-7.9	6.6	5.3-8.3	8.2	6.5-10.3	7.4	5.3-10.5	19.9	12.1-32.7
2013	6.2	5.7-6.6			6.1	4.9-7.6	6.4	5.1-8.0	8.0	6.4-10.1	7.2	5.1-10.2	19.4	11.8-31.9
2014	6.0	5.6-6.4			5.9	4.8-7.3	6.2	4.9-7.7	7.9	6.2-9.9	7.0	4.9-9.8	19.0	11.6-31.1
2015	5.8	5.4-6.2			5.7	4.6-7.1	6.0	4.7-7.5	7.7	6.1-9.7	6.7	4.7-9.5	18.5	11.3-30.4
2016	5.6	5.2-6.0			5.5	4.4-6.8	5.7	4.6-7.2	7.5	6.0-9.5	6.5	4.6-9.2	18.0	11.0-29.6
2017							5.6	4.4-7.0	7.4	5.8-9.3	6.3	4.4-8.9	17.6	10.7-28.9
2018									7.2	5.7-9.1	6.1	4.3-8.6	17.2	10.5-28.2
2019									7.0	5.6-8.9	5.9	4.1-8.3	16.7	10.2-27.5
2020									6.9	5.5-8.7	5.7	4.0-8.0	16.3	9.9-26.8

Notes: Fatality rate data for 2009 (presented in italics) are preliminary estimates of actual rates rather than predicted values. Predictions for NSW and Tas. have been determined using 1971-2009 rather than 1992-2009 data. Predictions for the other jurisdictions are based on 1992-2009 data.

## Discussion

Quantitative road safety targets have been advocated as a crucial component of effective road safety strategies [3], as empirical evidence suggests that such targets are correlated with objective achievements in road safety. Though the target of no more than 5.6 RTC fatalities per 100,000 was established using plausible estimates of the effects of a number of known measures and was described as challenging but realistic [59,60], the results of this analysis suggest that on the basis of past outcomes that the target may not be achieved nationally for a number of years. Progress towards the national target has been monitored since its implementation and reported on in biennial *National Road Safety Action Plans *by the Australian Transport Council [59-63]. In the plan for 2007 and 2008, various reasons for the slower than expected improvement were suggested. These included a greater than expected growth in VKT and in motorcycle and four-wheel-drive vehicles sales. Insufficient initiatives in speed management and road engineering, and increased sources of driver distraction (such as navigation systems, mobile phones, advertising billboards and traffic signs) were also thought to have played a role. Knowledge of factors that may be hindering progress is crucial if improvements in road safety are to continue.

The analysis also highlights the regional differences in the past trends in fatality rates and therefore also in the estimated time before the national target is reached in the respective jurisdictions. Given the variation in the factors that can affect road safety across the nation, this outcome is not entirely unexpected. Apart from differences in road safety measures, regulations, and law enforcement efforts, considerable diversity also exist in environmental conditions and the quality of the road network. For instance, while 42% of the length of roads (open to the public) in Australia (i.e., 337,000 of the 810,600 kms of road) was bitumen or concrete in 2004 (as opposed to gravel, crushed stone, other improved surface, or formed or cleared surface only), the percentage of such roads ranged from 95% in the ACT to just 29% in the NT [64]. Spatial differences in socio-demographic variables that can relate to road safety outcomes also exist. For instance, alcohol consumption has been the highest in the NT with an average annual consumption of 14.6 litres per adult (1991/92-1995/96) compared to the Australian average of 9.75 litres [65]. In addition, the NT's population was the youngest in Australia in 2009 with a median age 5.5 years less than the national median age [66]. Accordingly, the achievements of some of the states and territories provide cause for optimism but are less encouraging in others.

It is worth reiterating the limitations of the study as it is possible that if a structural model incorporating factors that affect road safety outcomes had been estimated rather than a pure time series model, different predictions may have resulted. However, the potential number of explanatory variables that would warrant inclusion, at least in an initial general specification of a causal/structural model, would be substantial. As the aim here was to use only recent trends in the fatality rates as a basis for the predictions, the number of available observations was necessarily limited. Thus, degrees of freedom issues would have arisen if a plausible structural model (that attempted to account for all possible influencing factors) had been specified. In support of the approach used, Elvik [52] has argued that time series models can describe long-terms trends in road safety outcomes nearly as well as structural models that employ various explanatory variables. However, he has also reported that different trend models that fit RTC data almost as well as each other can result in substantially different predictions of future outcomes [45,52].

The methods that were used to produce the results invoke an implicit assumption: that past trends will continue, essentially unchanged, in future years. While past trends are one means by which future outcomes can be predicted, whether or not they will continue unchanged can-not be predicted with certainty. While the forecasts presented here were based on models that met acceptable and standard statistical criteria, the results need to be interpreted with caution and in light of the methodological limitations we have highlighted.

Many factors influence RTC rates and the resulting fatality rates. Whether or not future safety targets will be met depends upon interactions between contributing causal factors and mitigation measures that are implemented to reduce the incidence and severity of RTCs that do occur. Encouragingly, in the past, certain interventions e.g., the introduction of compulsory seat belts lowered the impact of RTCs in a substantial and sustained fashion. The ongoing challenge for pubic health researchers is to identify similar trend-changing initiatives. In the absence of technological changes, these become increasingly difficult to achieve at the margin, given the advances that already have been made.

## Conclusions

This research has reviewed progress towards the RTC fatality target set that was for Australia in 2001 As sizeable gains have been achieved in most of the jurisdictions, the efforts of policy-makers, law enforcement agencies and the general public should be acknowledged. Encouragingly, preliminary data for 2010 also indicate that the downward trend in fatalities at a national level is continuing but the outcomes across the states and territories are mixed [67]. By disaggregating the analysis to the state and territory level thereby highlighting regional differences, the results allow a comparison of where the greatest improvements have been achieved and where revised efforts may be beneficial. Such information enables priorities to be reassessed and strategies to be revised (and would not have been ascertained if the analysis had only been conducted at a national level). The study also raises the challenging issue of reallocating resources. As the costs of achieving reductions in the road toll in each of the jurisdictions are likely to vary considerably, the question of how best to distribute national resources across the jurisdictions is by no means straightforward. The results of this analysis may provide useful information for policy-makers who are charged with developing further strategies to effectively reduce the road toll. They may also be used to stimulate a renewed interested in, and commitment to road safety amongst the general public, especially in jurisdictions where the public health burden from RTCs is known to be comparatively high.

## Competing interests

The Centre for National Research on Disability and Rehabilitation Medicine (CONROD) at The University of Queensland receives funding from the Motor Accident Insurance Commission (MAIC). The MAIC is a statutory body, established under the auspices of the Queensland (i.e., state) Treasury, to regulate compulsory third-party (personal injury, auto) markets in Queensland, Australia. MAIC had no role in the initiation of this study, the study design, data collection, analysis and interpretation, authorship and editing of this manuscript, nor in the decision to submit the manuscript for publication.

## Authors' contributions

All authors contributed to the conception and design of the study. SG was the chief investigator and collected the data, drafted the first manuscript and contributed to the data analysis. LBC made contributions to the data's interpretation and to revising the manuscript. SN made contributions to the statistical analysis and to revising the manuscript. All authors read and approved the final manuscript.

## Pre-publication history

The pre-publication history for this paper can be accessed here:

http://www.biomedcentral.com/1471-2458/11/270/prepub
